# Early initiation of breastfeeding, bottle feeding, and experiencing feeding challenges are associated with malnutrition

**DOI:** 10.1002/fsn3.3472

**Published:** 2023-06-12

**Authors:** Humphrey Garti, Mohammed Bukari, Anthony Wemakor

**Affiliations:** ^1^ Department of Nutritional Sciences, School of Allied Health Sciences University for Development Studies Tamale Ghana

**Keywords:** breastfeeding, child feeding, complementary feeding, Ghana, indicators, practices, undernutrition

## Abstract

Malnutrition remains a public health concern amidst low proportions of the core infant and young child feeding (IYCF) practices, yet, data on specific child feeding practices that are associated with undernutrition are rare. Hence, this study sought to assess child feeding practices and their association with undernutrition among young children. An analytical cross‐sectional design was used among mothers/caregivers with children aged 6–23 months, attending child welfare clinics in Techiman municipality, Ghana. Simple random sampling was used to select 8 health facilities, and 403 participants were selected from those facilities using proportional stratification. A 24‐h dietary recall based on seven food groups was used to collect data on children's dietary intake and used to derive WHO child feeding indicators. The length, weight, and age of children were taken and used to compute anthropometric *z*‐scores. The proportions of children who met their minimum dietary diversity (MDD), minimum meal frequency (MMF), and minimum acceptable diet (MAD) were 44%, 56%, and 36% respectively. Children 6–8 months [AOR=2.24, CI (1.037–4.841); *p* = .04] and 9‐11 months [AOR=2.47, CI (1.096–5.573); *p* = .029], those who were not breastfed within the first hour of delivery [AOR = 3.56, CI (1.833–6.912), *p* < .001], and those who were bottle fed [AOR = 2.87, CI (1.374–5.973); *p* = .005] were more likely to be wasted. Children 6–8 months [AOR = 0.29, CI (0.126–0.672); *p* = .004] and 9–11 months [AOR = 0.24, CI (0.104–0.544); *p* = .001] and those who experienced feeding challenges [AOR = 0.52, CI (0.301–0.905); *p* = 0.021] were protected against stunting. The percentages of children who met their MDD, MMF, and MAD were low and not associated with undernutrition. Early initiation of breastfeeding and bottle feeding were associated with acute malnutrition and experiencing feeding challenges was associated with chronic malnutrition. Promoting appropriate child feeding practices can reduce the risk of undernutrition.

## Graphical abstract

This study sought to assess child feeding practices and their association with undernutrition among children 6–23 months old. We found that early initiation of breastfeeding and bottle feeding were associated with acute malnutrition and experiencing feeding challenges was associated with stunting. Child feeding indicators such as minimum dietary diversity, minimum meal frequency, and minimum acceptable diet were not associated with malnutrition. The findings could help improve existing programs at child welfare clinics.

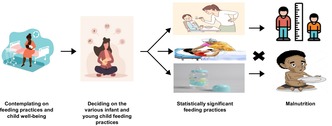

## 1 INTRODUCTION

Complementary feeding refers to the timely introduction of safe and nutritious foods in addition to breastfeeding typically from 6 to 23 months of age (Bhutta et al., 2013). A survey in low‐income countries showed that eating diverse diets lowers the chances of being stunted and/or underweight, and minimum meal frequency reduces the chances of being underweight (Bhutta et al., 2013). In Ghana, the proportion of children aged 6–23 months who met the three core infant and young child feeding (IYCF) practices was only 13% (GSS et al., 2015). Another study found that the proportions of children aged 6–23 months who met the minimum meal frequency (MMF) and minimum dietary diversity (MDD) were 46.0% and 51.4% respectively (Issaka et al., 2015).

Only 29% of infants globally meet their MDD (Bégin & Aguayo, 2017). According to Lutter et al. (2011), on the global burden of malnutrition and the highlight of data on child feeding practices, less than 30% of children aged 6–23 months achieved the MDD. A study by Kassa et al. (2016) showed that only 18.8% of infants in Benin met their MDD. Similarly, a study by Mekonnen et al. (2017) reported that 27.3% of children met their MDD with most of them consuming grains (84.6%) while just a few consumed fish (9.7%) and 1.1% consumed iron‐rich foods (liver).

Globally, only 52% of children between the ages of 6–23 months met their MMF (White et al., 2017). Kassa et al. (2016) found that 67.3% of children met their MMF. Vitta et al. (2016) also reported that 70.3% of children between the ages of 6–23 months met their MMF in Tanzania which is higher than most studies. However, some studies in Ghana have found lower proportions of children receiving the recommended frequency of foods compared to the global prevalence, that is, 29% for Ga West Municipality and 40% for Tema Municipality (Agbozo et al., 2016; Bentil et al., 2016).

Most studies have shown that few children are able to meet both MDD and MMF, and do not meet their MAD and optimal complementary feeding with a global prevalence of MAD representing only 15.9% (White et al., 2017). Lower prevalences have been recorded in some African countries 12.3%, 7.3%, and 21.1%, for Benin, Nigeria, and Ethiopia respectively (Kassa et al., 2016; Mekonnen et al., 2017; Udoh & Amodu, 2016). Since minimum acceptable diet is directly associated with optimal complementary feeding, the findings above show that complementary feeding is a challenge in developing countries and progress towards meeting the standard for optimal complementary feeding is slow. These underscore the relevance of examining current feeding practices which may be critical for achieving optimal nutritional status, in addition, malnutrition is a public health concern in Ghana (GSS et al., 2015). Hence, there is the need to identify and promote feeding practices that have the potential to improve child nutritional status, yet, there is little data on the relationship between child feeding practices and nutritional status in the middle belt of Ghana, specifically, Techiman municipality. Hence, this study sought to assess child feeding practices and their association with undernutrition among children 6 to 23 months old in the Techiman municipality of Ghana.

### 1.1 Key message

- One‐third of mothers experienced feeding challenges when feeding children, and one out of every six children was bottle fed. Feeding challenges were categorized into three: (1) Food insecurity; (2) Vomiting and refusal to/after eat(ing); and (3) Inadequate knowledge on appropriate child feeding practices and lack of time.
- Early initiation of breastfeeding and bottle feeding were associated with acute malnutrition and experiencing feeding challenges was associated with stunting. Child feeding indicators such as minimum dietary diversity, minimum meal frequency, and minimum acceptable diet were not associated with malnutrition.
- The findings could be relevant for nutrition educators and nutrition programmers, as efforts could be tailored towards improving infant and young child feeding practices and strengthening existing programs such as growth monitoring, and promotion activities in child welfare clinics.

## 2 METHODS

### 2.1 Study area

The study was conducted in the Brong Ahafo region (which has been recently divided into Bono East and Ahafo regions) which has two farming seasons. It lies between the northern and southern part of Ghana, mostly regarded as the middle belt. The Brong Ahafo region is one of the major producers of cocoa and timber in the forest zone of Ghana.

### 2.2 Design, target population, and sampling

The analytical cross‐sectional design was used among mothers/caregivers with children, aged 6–23 months, attending child welfare clinics in Techiman municipality, Ghana. Eligible study participants who declined consent and sick children were excluded from the study. Decision on consent was taken by mothers/caregivers; sickness was defined by observation, self–report of mothers/caregivers, consultation with healthcare personnel and child health records booklets. A sample size of 403 was obtained using the formula for sample size determination with single proportion (Hawkins, 1989); an assumed prevalence of 50% for undernutrition, a critical value of 1.96 at 95% confidence level, and a 0.05 margin of error. Simple random sampling was used to select 8 health facilities (Grace Hospital, Opoku Agyemang Hospital, Holy Family Hospital, Tanoso Health Centre, Bamire Health Centre, Mt. Olive Hospital, Atabuoso Health Centre, and Akrufi Health Centre) from a total of 14 health facilities in the municipality, and the 403 participants were selected from these facilities using proportional stratification.

### 2.3 Data collection tools and procedures

#### 2.3.1 Assessment of child feeding indicators

A single 24‐h recall was used to collect data on children's dietary intake. Mothers were asked to recall foods that were given to their children in the last 24 h, and the various foods mentioned were classified into seven food groups; Cereals; Legumes and Nuts; Dairy products; Flesh foods; Eggs; Vitamin A rich fruits and vegetables; Other fruits and vegetables. Children were given a score of 1 for consuming food from a food group and 0 for otherwise. The sum of the individual food groups gave dietary diversity score, the maximum score for a child was 7 and the minimum score was 0 when a child did not consume from any of the food groups. Children whose dietary diversity scores were ≥4 were considered to have met their MDD; children who were 6–8 months old or 9–12 months old, were still breastfed, and had eaten a solid or semi‐solid food 2 and 3 times in a day respectively were considered to have met their MMF. Children aged 6–23 months who were not breastfed and had eaten a solid or semi‐solid food 4 times in a day were also considered to have met their MMF. Children who met both MDD and MMF were considered to have met their MAD. All these computations were performed in line with WHO guidelines (WHO, 2010). Early initiation of breastfeeding, bottle feeding, feeding challenges, introduction of solid or semi‐solid foods at 6 months, continued breastfeeding at 1 year, and giving colostrum to the child were also assessed by asking mothers to respond to yes or no questions on the aforementioned indicators. For those who said they experienced feeding challenges, an open‐ended question (what challenges did you experience?) was used to probe in order to understand the challenges they experienced.

#### 2.3.2 Assessment of nutritional status

Length of each child was taken with a height measuring board and weight was measured using an electronic weighing scale (SECA). All measurements were recorded to the nearest 0.1 cm or 0.1 kg for height and weight respectively. Ages of children were confirmed from records on date of births in child health record booklets. The height/length, weight, and age were used to compute anthropometric *z*‐scores. Anthropometric *z*‐scores of <−2 standard deviations (−2SD) from the median of the WHO Growth Standards were defined as stunting, wasting, and underweight (WHO, 2006). Data on the kind of feeding challenges participants experienced were analyzed using thematic analysis.

### 2.4 Study variables

#### 2.4.1 Independent variables

The independent variables were child's age, place of delivery, and child feeding practices. The child feeding practices measured in this study were initiation of breastfeeding, colostrum use, continued breastfeeding at 1 year, foods introduced before breastmilk, introduction of solid and semi‐solid foods at 6 months, feeding challenges experienced by mothers, bottle feeding, MDD, MMF, and MAD.

#### 2.4.2 Dependent variables

The dependent variable was undernutrition which was measured using stunting, wasting, and underweight.

### 2.5 Data analysis

Data were entered, cleaned, and analyzed using Statistical Package for the Social Sciences (SPSS), version 21 (IBM Inc.). Microsoft Excel 2014 and WHO anthro software were used to calculate weight–for–height z–scores (WHZ), weight–for–age z–scores (WAZ), and length–for–age z–scores (LAZ). Categorical variables were presented as frequencies and percentages, and continuous variables were presented as means and standard deviations. First‐line analysis was performed using Chi‐square/Fisher's Exact test to identity child feeding indicators that affected malnutrition (See Table S1). Variables with *p*‐values <.2 were fitted in the multiple logistic regression models (Hosmer Jr et al., 2013; Sitotaw et al., 2019) to determine their independent contributions to children's nutritional status. The goodness of fit of the models was assessed using Hosmer & Lemeshow test; stunting model (*X* ^2^ = 6.32, *p* = .61), wasting model (*X* ^2^ = 1.66, *p* = .80), and underweight model (*X* ^2^ = 2.18, *p* = .70), which indicated acceptable goodness of fit. Statistical significance was set at *p* < .05 for the logistic regression analyses.

## 3 RESULTS

### 3.1 Socio‐demographic characteristics of respondents

A total of 403 participants were interviewed. About 46% of children were between 12 and 23 months old, 53% of them were males and 83% of them were born in health facilities. More than half of the mothers (52.6%) had low educational background and 8 out of every 10 mothers were gainfully employed (Table 1).

**TABLE 1:** Socio‐demographic characteristics of participants.

Characteristic	Frequency	Percentage
Age of children (months)		
6–8	133	33
9–11	85	21.1
12–23	185	45.9
Sex of child		
Female	190	47.1
Male	213	52.9
Educational level of mothers		
None (No formal education)	43	10.7
Low (Primary and junior high school)	212	52.6
High (Senior high school, vocational school, and tertiary)	148	36.7
Employment status of mothers		
Gainfully employed	337	83.6
Not employed	66	16.4
Place of delivery		
Home	68	16.9
Health facility	335	83.1

### 3.2 Distribution of dietary practices and nutritional status by age

Children who met their MDD were 44%, out of this percentage, 65% were 12 to 23 months old (Table 2). Children who met their MMF were 56%, and six out of every ten of them were 12 to 23 months old. Thirty‐six percent of them met their MAD and 25% of these children were 9 to 11 years old. Twenty‐one percent (20.6%) of all children were stunted and 69% of the stunted children were 12 to 23 months old, four out of ten children who were wasted were 6 to 8 months old (11% of all children were wasted) and 15% of underweight children were 9 to 11 months old (10% of all children were underweight) (Table 2).

**TABLE 2:** Distribution of dietary practices and nutritional status by age.

Characteristics	Age of child in months			
	*N*	6–8	9–11	12–23
Minimum dietary diversity (MDD) (≥4 food groups)	176	15 (8.5%)	47 (26.7%)	114 (64.8%)
Minimum meal frequency (MMF)	224	45 (20.1%)	49 (21.9%)	130 (58.0%)
Minimum acceptable diet (MAD)	145	13 (9%)	36 (24.8%)	96 (66.2%)
Stunting	83	17 (20.5%)	8 (9.6%)	58 (69.9%)
Wasting	46	18 (39.1%)	14 (30.4%)	14 (30.4%)
Underweight	39	19 (48.7%)	6 (15.4%)	14 (35.9%)

### 3.3 Feeding characteristics of children

The percentage of children that were fed within the first hour of birth was 80.4%, less than one percent (0.7%) of them were not given colostrum and nine out of every ten (93.5%) were still breastfed at 1 year. Nearly all (98%) children were not given any food before breastmilk and more than three‐quarter (79.7%) of them started eating solid and semi‐solid foods at 6 months old. One‐third of mothers/caregivers experienced feeding challenges when feeding their children and 84% of children were not bottle fed (Table 3). Feedback from probing on the feeding challenges experienced were grouped into three: (1) Food insecurity; (2) Vomiting and refusal to eat/ vomiting after eating; and (3) Inadequate knowledge on appropriate child feeding practices and lack of time.

**TABLE 3:** Feeding characteristics of children.

Characteristics	Frequency	Percentage
Early initiation of breastfeeding		
Within 1 h of delivery	324	80.4
After 1 h of delivery	79	19.6
What was done with colostrum?		
Gave it to baby	400	99.3
Discarded it	3	0.7
Continued breastfeeding at 1 year		
Yes	377	93.5
No	26	6.5
Food introduced before breastmilk		
Nothing	395	98
Infant formula and other foods	8	2
Introduction of solid and semi‐solid food at 6 months		
Yes	321	79.7
No	82	20.3
Experienced feeding challenges		
Yes	269	66.8
No	134	33.2
Bottle feeding		
Yes	65	16.1
No	338	83.9

### 3.4 Multivariable analysis of child feeding factors and nutrition status of children

In Table 4, the logistic regression analysis revealed that children 6 to 8 months old were 71% less likely to be stunted compared to children 12to 23 months old [AOR = 0.29, 95% CI (0.126–0.672); *p* = .004]. Also, children 9 to 11 months old [AOR=0.24, 95% CI (0.104–0.544); *p* = .001] were protected against stunting as compared to their counterparts who were 12 to 23 months old. Experiencing feeding challenges was protective against stunting [AOR = 0.52, 95% CI (0.301–0.905); *p* = .021]. Children 6 to 8 months old were 2.24 times more likely to be wasted compared to children 12 to 23 months old [AOR = 2.24, 95% CI (1.037–4.841); *p* = .040]. Children 9 to 11 months old [AOR=2.47, 95% CI (1.096–5.573); *p* = 0.029] were also more likely to be wasted compared to children 12 to 23 months old. Children who were given breastmilk after the first hour of delivery were 3.6 times more likely to be wasted compared to those who were given breastmilk within the first hour of delivery [AOR = 3.56, 95% CI (1.833–6.912); *p* = <.001]. Children who were introduced to bottle feeding were 2.87 times more likely to be wasted compared to those who were not introduced to bottle feeding [AOR = 2.87, 95% CI (1.374–5.973); *p* = .005].

**TABLE 4:** Multivariable analysis of child feeding factors and nutrition status of children.

Variables	*p*‐Value	Adjusted odds ratio (AOR)	95% CI for AOR	
			Lower	Upper
Stunting				
Age of children (reference; 12–23)				
6–8	.004	0.291	0.126	0.672
9–11	.001	0.238	0.104	0.544
Place of delivery: home (reference; health facility)	.064	1.838	0.964	3.501
Early initiation of breastfeeding: after first hour of delivery (reference; within first hour of delivery)	.253	0.653	0.314	1.357
Continued breastfeeding at 1 year: no (reference; yes)	.094	2.228	0.873	5.683
Introduce solid and semi‐solid food at 6 months: no (reference; yes)	.583	0.742	0.256	2.150
Experienced feeding challenges: yes (reference; no)	.021	0.522	0.301	0.905
Bottle feeding: yes (reference; no)	.073	0.476	0.212	1.072
Minimum meal frequency (MMF): no (reference; yes)	.543	1.219	0.643	2.311
Constant	.179	0.677		
Wasting				
Age of children (reference; 12–23)				
6–8	.040	2.240	1.037	4.841
9–11	.029	2.471	1.096	5.573
Early initiation of breastfeeding: after first hour of delivery (reference; within first hour of delivery)	<.001	3.560	1.833	6.912
Bottle feeding: yes (reference; no)	.005	2.865	1.374	5.973
Constant	<.001	0.044		
Underweight				
Age of children (reference; 12–23)	.778			
6–8	.519	1.364	0.531	3.502
9–11	.932	0.958	0.353	2.596
Early initiation of breastfeeding: after first hour of delivery (reference; within first hour of delivery)	.095	1.917	0.892	4.120
Introduce solid and semi‐solid food at 6 months: no (reference; yes)	.134	2.086	0.797	5.460
Constant	.000	0.067		

## 4 DISCUSSION

The study sought to investigate the association between child feeding practices and undernutrition, specifically, stunting, wasting, and underweight, and found that not giving breastmilk to children within first hour of delivery and practicing bottle feeding were positively associated with wasting. The study also revealed that younger child age and experiencing feeding challenges were protective against stunting.

In the current study, the percentage of children that was bottle fed was almost 18% and 43% higher than the number of children who were bottle fed in studies by Jemide et al. (2016) and Abeway et al. (2018) in Nigeria and Ethiopia respectively. Degefa et al. (2019) also reported bottle feeding prevalence of 35.1% in Burayu, Ethiopia. The differences in these findings may be because of tailored health messages on dangers of bottle feeding given to mothers in the current study during growth monitoring and promotion service at child welfare clinics. The percentage of children introduced to breastmilk within the first hour of birth was 80% in the current study which confirmed the findings of Abeway et al. (2018) but much higher than the average figure reported for urban and rural communities in Bangladesh (51%) (Sheikh et al., 2020). The current study also reported a high percentage of children being introduced to solid and semi‐solid foods at 6 months, this was in line with the finding of Jemide et al. (2016). Sheikh et al. (2020) also reported that 65% of children were introduced to solid and semi‐solid foods in Bangladesh.

The percentage of children that met their MAD was higher than other studies conducted in Ghana (Saaka et al., 2015) and Nigeria (Jemide et al., 2016). The variation in findings may be because health workers in the current study became more experienced and savvy with nutrition counseling in the course of providing growth monitoring and promotion services at child welfare clinics over time. However, the findings of Saaka et al. (2015) on proportion of children meeting their MMF was similar to the finding of the current study. This implies that more children would meet their energy requirements since MMF is a proxy for measuring energy intake (Mekonnen et al., 2017).

The current study did not show any association between the WHO main child feeding indicators; MDD, MMF, and MAD, and malnutrition in the multivariable logistic regression analyses. Our findings were similar to studies from Cambodia (Reinbott et al., 2015) and Ethiopia (Tessema et al., 2013). Additionally, another study in Ghana (Saaka et al., 2015) did not show any association between minimum dietary diversity, minimum meal frequency, and stunting. On the contrary, some studies indicated increased risk for stunting and underweight among children who did not meet their minimum dietary diversity (Fekadu et al., 2015; Khamis et al., 2019). Other studies by Kimwele and Ochola (2017) and Girma et al. (2019) showed association between minimum meal frequency and dietary diversity as well as all three forms of malnutrition. Similarly, in Nigeria, increased odds of underweight and stunting among children who did not meet their minimum dietary diversity were noted (Udoh & Amodu, 2016). Saaka et al. (2015) suggested that the lack of association between stunting and MDD, MMF, and MAD may be because the feeding indicators are not sensitive to chronic malnutrition.

Child feeding indicators such as early initiation of breastfeeding and bottle feeding remained significantly associated with wasting after multivariable logistic regression analysis. The current study established that bottle feeding increased the risk of wasting compared to children who were not bottle fed. This confirm findings from other studies that suggested increased odds of wasting among bottle fed children (Ejigu et al., 2018; Liben et al., 2016). Our study also showed an increased risk of wasting among children who were not breastfed within the first hour of birth. Marriott et al. (2012)reported significant association between early initiation of breastfeeding and protection against underweight; their study also showed that children face a greater risk of underweight if breast feeding does not continue to between 12 and 15 months. Underweight may reflect wasting or stunting (Prendergast, 2015), hence, it is important to adhere to WHO recommendations on child feeding to avoid acute and subsequent malnutrition. Other studies also showed increased risk of stunting among children who were not breastfed within the first hour of birth (Kismul et al., 2018; Muchina & Waithaka, 2010; Shine et al., 2017; Simanjuntak et al., 2018).

Interestingly, the findings of the current study indicate that children of mothers who experienced feeding challenges were protected against stunting. It would make sense to suggest that these mothers probably visited health facilities and nutrition experts more, and the present observation could be a reflection of knowledge and skills they acquired from health personnel and child feeding support groups on how to overcome such challenges. Child feeding indicators; MMF, introduction of solid and semi‐solid foods at 6 months, and continued breastfeeding were significantly associated with malnutrition in bivariate analysis but not in the multivariable analysis. This may suggest that other important factors are required in addition to the aforementioned feeding indicators to achieve normal nutritional status.

Considering the total number of children who were appropriately fed in this study, and the high prevalence of malnutrition, there is the need for more efforts in the area of social behavior change communication among mothers and pregnant women, social behavior change materials such as posters and calendars could be made more readily available than it is currently. In addition, health workers or community health volunteers could make regular follow‐ups to ensure that mothers are implementing all that they learn at child welfare clinics. This has the potential to enhance uptake of key nutrition messages and also improve access to child welfare clinic services which has been suggested to improve infant and young child feeding (Aborigo et al., 2012; Apanga, 2015; Otoo et al., 2009). As has been shown in the current study, inappropriate child feeding practices namely giving the child breastmilk after the first hour of delivery and bottle feeding increase the risk of undernutrition. Therefore, promoting appropriate child feeding practices particularly early initiation of breastfeeding can reduce the risk of undernutrition.

This study had some limitations. For instance, its cross‐sectional nature made it impossible to determine the effect of child feeding practiceson malnutrition. Also, mothers/caregivers reliance on memory during 24‐h recall could have introduced some recall bias but several probing techniques were used to minimise recall bias. That notwithstanding, we believe the current study establishes that child feeding practices such as early initiation of breastfeeding, bottle feeding, and mothers/caregivers experience of feeding challenges are associated with malnutrition among children. The findings could be relevant for nutrition educators and nutrition programmers, as efforts could be tailored towards improving infant and young child feeding practices and strengthening existing programs such as growth monitoring and promotion activities in child welfare clinics.

## 5 CONCLUSION

The proportions of children who meet their MDD, MMF, and MAD were low but not associated with undernutrition. Not practicing early initiation of breastfeeding and bottle feeding were associated with acute malnutrition, and experiencing feeding challenges was associated with chronic malnutrition. Also, younger child age was a protective factor for stunting but a risk factor for wasting. Promoting appropriate child feeding practices can reduce the risk of undernutrition.

## AUTHOR CONTRIBUTIONS

**Humphrey Garti:** Conceptualization (equal); data curation (equal); formal analysis (supporting); investigation (equal); methodology (equal); project administration (equal); supervision (lead); writing – original draft (supporting); writing – review and editing (equal). **Mohammed Bukari:** Conceptualization (equal); data curation (equal); formal analysis (lead); investigation (equal); methodology (equal); project administration (equal); software (lead); supervision (supporting); writing – original draft (lead); writing – review and editing (equal). **Anthony Wemakor:** Conceptualization (equal); data curation (equal); formal analysis (equal); investigation (equal); methodology (equal); project administration (equal); software (equal); supervision (equal); writing – original draft (supporting); writing – review and editing (equal).

## CONFLICT OF INTEREST STATEMENT

The authors declare they have no competing interests.

## ETHICAL STATEMENT

Ethical approval was obtained from the Committee on Human Research, Publication and Ethics, KNUST, College of Health Sciences, Kumasi with reference number CHRPE/AP/152/22 and permission was obtained from the Municipal Health Directorate, Techiman.

## CONSENT TO PARTICIPATE

Informed consent was obtained from mothers/caregivers before the study has begun. Participants were guaranteed privacy and confidentiality during data collection. They were informed that they could wthdraw or decline to respond at any point in the study.

## Supporting information


Table S1

## Data Availability

The dataset supporting the findings of this study could be obtained from the corresponding author upon reasonable request.

## References

[fsn33472-bib-0001] Abeway, S. , Gebremichael, B. , Murugan, R. , Assefa, M. , & Adinew, Y. M. (2018). Stunting and its determinants among children aged 6–59 months in northern Ethiopia: A cross‐sectional study. Journal of Nutrition and Metabolism, 2018, 1078480. 30046469 10.1155/2018/1078480 PMC6036796

[fsn33472-bib-0002] Aborigo, R. A. , Moyer, C. A. , Rominski, S. , Adongo, P. , Williams, J. , Logonia, G. , Affah, G. , Hodgson, A. , & Engmann, C. (2012). Infant nutrition in the first seven days of life in rural northern Ghana. BMC Pregnancy and Childbirth, 12(1), 1–10. 22857600 10.1186/1471-2393-12-76 PMC3490996

[fsn33472-bib-0003] Agbozo, F. , Colecraft, E. , & Ellahi, B. (2016). Impact of type of child growth intervention program on caregivers' child feeding knowledge and practices: A comparative study in Ga West Municipality, Ghana. Food Science & Nutrition, 4(4), 562–572. 27386106 10.1002/fsn3.318 PMC4930500

[fsn33472-bib-0004] Apanga, P. A. (2015). Infant and young child feeding in children under‐5 years in Ghana: Key strategy to childhood development. Journal of Advances in Medicine and Medical Research, 8, 343–347.

[fsn33472-bib-0005] Bégin, F. , & Aguayo, V. M. (2017). First foods: Why improving young children's diets matter. Maternal & Child Nutrition, 13, e12528. 29032619 10.1111/mcn.12528 PMC6866215

[fsn33472-bib-0006] Bentil, H. J. , Steiner‐Asiedu, M. , & Lartey, A. (2016). Comparison of the complementary feeding practices between mothers with twins and mothers with singletons. The Pan African Medical Journal, 24, 52. 27642393 10.11604/pamj.2016.24.52.8290 PMC5012738

[fsn33472-bib-0007] Bhutta, Z. A. , Das, J. K. , Rizvi, A. , Gaffey, M. F. , Walker, N. , Horton, S. , Webb, P. , Lartey, A. , Black, R. E. , Lancet Nutrition Interventions Review Group , & the Maternal and Child Nutrition Study Group . (2013). Evidence‐based interventions for improvement of maternal and child nutrition: What can be done and at what cost? Lancet, 382(9890), 452–477. 23746776 10.1016/S0140-6736(13)60996-4

[fsn33472-bib-0008] Degefa, N. , Tadesse, H. , Aga, F. , & Yeheyis, T. (2019). Sick child feeding practice and associated factors among mothers of children less than 24 months old, in Burayu town, Ethiopia. International Journal of Pediatrics, 2019, 3293516. 31929806 10.1155/2019/3293516 PMC6942812

[fsn33472-bib-0009] Ejigu, B. , Legesse, T. G. , & Chercos, D. H. (2018). Prevalence and associated factors of malnutrition among children aged 6–59 months in Addi Harush Eritrean refugees camp, Tigray Region, North Ethiopia. Journal of Pharmacy and Nutrition Sciences, 7, 164–171.

[fsn33472-bib-0010] Fekadu, Y. , Mesfin, A. , Haile, D. , & Stoecker, B. J. (2015). Factors associated with nutritional status of infants and young children in Somali Region, Ethiopia: A cross‐sectional study. BMC Public Health, 15(1), 846. 26330081 10.1186/s12889-015-2190-7 PMC4557759

[fsn33472-bib-0011] Girma, A. , Woldie, H. , Mekonnen, F. A. , Gonete, K. A. , & Sisay, M. (2019). Undernutrition and associated factors among urban children aged 24–59 months in Northwest Ethiopia: A community based cross sectional study. BMC Pediatrics, 19(1), 214. 31255179 10.1186/s12887-019-1595-3 PMC6599324

[fsn33472-bib-0012] GSS , GHS , & ICF Macro . (2015). Ghana demographic and health survey 2014. Ghana Statistical Service, Ghana Health Service, and ICF International.

[fsn33472-bib-0013] Hawkins, D. (1989). Using U statistics to derive the asymptotic distribution of Fisher's Z statistic. The American Statistician, 43(4), 235–237.

[fsn33472-bib-0014] Hosmer, D. W., Jr. , Lemeshow, S. , & Sturdivant, R. X. (2013). Applied logistic regression (Vol. 398). John Wiley & Sons.

[fsn33472-bib-0015] Issaka, A. I. , Agho, K. E. , Burns, P. , Page, A. , & Dibley, M. J. (2015). Determinants of inadequate complementary feeding practices among children aged 6–23 months in Ghana. Public Health Nutrition, 18(4), 669–678. 24844532 10.1017/S1368980014000834 PMC10271301

[fsn33472-bib-0016] Jemide, J. O. , Ene‐Obong, H. N. , Edet, E. E. , & Udoh, E. E. (2016). Association of maternal nutrition knowledge and child feeding practices with nutritional status of children in Calabar South Local Government Area, Cross River State, Nigeria. International Journal of Home Science, 2(1), 293–298.

[fsn33472-bib-0017] Kassa, T. , Meshesha, B. , Haji, Y. , & Ebrahim, J. (2016). Appropriate complementary feeding practices and associated factors among mothers of children age 6–23 months in Southern Ethiopia, 2015. BMC Pediatrics, 16(1), 131. 27542833 10.1186/s12887-016-0675-x PMC4992197

[fsn33472-bib-0018] Khamis, A. G. , Mwanri, A. W. , Ntwenya, J. E. , & Kreppel, K. (2019). The influence of dietary diversity on the nutritional status of children between 6 and 23 months of age in Tanzania. BMC Pediatrics, 19(1), 518. 31881999 10.1186/s12887-019-1897-5 PMC6935228

[fsn33472-bib-0019] Kimwele, A. , & Ochola, S. (2017). Complementary feeding and the nutritional status of children 6–23 months attending Kahawa. West Public Health Center.

[fsn33472-bib-0020] Kismul, H. , Acharya, P. , Mapatano, M. A. , & Hatløy, A. (2018). Determinants of childhood stunting in the Democratic Republic of Congo: Further analysis of Demographic and Health Survey 2013–14. BMC Public Health, 18(1), 74. 10.1186/s12889-017-4621-0 PMC5540220 28764669

[fsn33472-bib-0021] Liben, M. L. , Abuhay, T. , & Haile, Y. (2016). Determinants of child malnutrition among agro pastorals in northeastern Ethiopia: A cross‐sectional study. Health Science Journal, 10(4), 1.

[fsn33472-bib-0022] Lutter, C. K. , Daelmans, B. M. , de Onis, M. , Kothari, M. T. , Ruel, M. T. , Arimond, M. , Deitchler, M. , Dewey, K. G. , Blössner, M. , & Borghi, E. (2011). Undernutrition, poor feeding practices, and low coverage of key nutrition interventions. Pediatrics, 128(6), e1418–e1427. 22065267 10.1542/peds.2011-1392

[fsn33472-bib-0023] Marriott, B. P. , White, A. , Hadden, L. , Davies, J. C. , & Wallingford, J. C. (2012). World Health Organization (WHO) infant and young child feeding indicators: Associations with growth measures in 14 low‐income countries. Maternal & Child Nutrition, 8(3), 354–370. 22171937 10.1111/j.1740-8709.2011.00380.x PMC6860880

[fsn33472-bib-0024] Mekonnen, T. C. , Workie, S. B. , Yimer, T. M. , & Mersha, W. F. (2017). Meal frequency and dietary diversity feeding practices among children 6–23 months of age in Wolaita Sodo town, Southern Ethiopia. Journal of Health, Population and Nutrition, 36(1), 18. 28526058 10.1186/s41043-017-0097-x PMC5437677

[fsn33472-bib-0025] Muchina, E. , & Waithaka, P. (2010). Relationship between breastfeeding practices and nutritional status of children aged 0‐24 months in Nairobi, Kenya. African Journal of Food, Agriculture, Nutrition and Development, 10(4), 2358–2378.

[fsn33472-bib-0026] Otoo, G. E. , Lartey, A. A. , & Pérez‐Escamilla, R. (2009). Perceived incentives and barriers to exclusive breastfeeding among periurban Ghanaian women. Journal of Human Lactation, 25(1), 34–41. 18971507 10.1177/0890334408325072 PMC4048713

[fsn33472-bib-0027] Prendergast, A. J. (2015). Malnutrition and vaccination in developing countries. Philosophical Transactions of the Royal Society B: Biological Sciences, 370(1671), 20140141. 10.1098/rstb.2014.0141 PMC4527386 25964453

[fsn33472-bib-0028] Reinbott, A. , Kuchenbecker, J. , Herrmann, J. , Jordan, I. , Muehlhoff, E. , Kevanna, O. , & Krawinkel, M. (2015). A child feeding index is superior to WHO IYCF indicators in explaining length‐for‐age Z‐scores of young children in rural Cambodia. Paediatrics and International Child Health, 35(2), 124–134. 10.1179/2046905514Y.0000000155 25226288 PMC4462840

[fsn33472-bib-0029] Saaka, M. , Wemakor, A. , Abizari, A.‐R. , & Aryee, P. (2015). How well do WHO complementary feeding indicators relate to nutritional status of children aged 6–23 months in rural northern Ghana? BMC Public Health, 15(1), 1157. 26596246 10.1186/s12889-015-2494-7 PMC4656186

[fsn33472-bib-0030] Sheikh, N. , Akram, R. , Ali, N. , Haque, S. R. , Tisha, S. , Mahumud, R. A. , Sarker, A. R. , & Sultana, M. (2020). Infant and young child feeding practice, dietary diversity, associated predictors, and child health outcomes in Bangladesh. Journal of Child Health Care, 24(2), 260–273. 31159554 10.1177/1367493519852486

[fsn33472-bib-0031] Shine, S. , Tadesse, F. , Shiferaw, Z. , Mideksa, L. , & Seifu, W. (2017). Prevalence and associated factors of stunting among 6‐59 months children in pastoral community of Korahay Zone, Somali Regional State, Ethiopia 2016. Journal of Nutritional Disorders & Therapy, 7(1), 2161‐0509.1000208.

[fsn33472-bib-0032] Simanjuntak, B. Y. , Haya, M. , Suryani, D. , & Ahmad, C. A. (2018). Early inititation of breastfeeding and vitamin a supplementation with nutritional status of children aged 6‐59 months. Kesmas: National Public Health Journal, 12(3), 107–113.

[fsn33472-bib-0033] Sitotaw, B. , Mekuriaw, H. , & Damtie, D. (2019). Prevalence of intestinal parasitic infections and associated risk factors among Jawi primary school children, Jawi town, north‐west Ethiopia. BMC Infectious Diseases, 19(1), 341. 10.1186/s12879-019-3971-x 31023271 PMC6485161

[fsn33472-bib-0034] Tessema, M. , Belachew, T. , & Ersino, G. (2013). Feeding patterns and stunting during early childhood in rural communities of Sidama, South Ethiopia. Pan African Medical Journal, 14(1), 75. 23646211 10.11604/pamj.2013.14.75.1630 PMC3641921

[fsn33472-bib-0035] Udoh, E. E. , & Amodu, O. K. (2016). Complementary feeding practices among mothers and nutritional status of infants in Akpabuyo Area, Cross River State Nigeria. SpringerPlus, 5(1), 2073. 10.1186/s40064-016-3751-7 28018781 PMC5138178

[fsn33472-bib-0036] Vitta, B. S. , Benjamin, M. , Pries, A. M. , Champeny, M. , Zehner, E. , & Huffman, S. L. (2016). Infant and young child feeding practices among children under 2 years of age and maternal exposure to infant and young child feeding messages and promotions in Dar es Salaam, Tanzania. Maternal & Child Nutrition, 12, 77–90. 27061958 10.1111/mcn.12292 PMC5071773

[fsn33472-bib-0037] White, J. M. , Bégin, F. , Kumapley, R. , Murray, C. , & Krasevec, J. (2017). Complementary feeding practices: Current global and regional estimates. Maternal & Child Nutrition, 13, e12505. 29032623 10.1111/mcn.12505 PMC6865887

[fsn33472-bib-0038] WHO . (2006). WHO Multicentre Growth Reference Study Group: WHO child growth standards: Length/height‐for‐age, weight‐for‐age, weight‐for‐length, weight‐for‐height and body mass index‐for‐age: Methods and development. WHO, 2007.

[fsn33472-bib-0039] WHO . (2010). Indicators for assessing infant and young child feeding practices: Part 2: Measurement .

