# Supplementary material for: Early initiation of breastfeeding, bottle feeding, and experiencing feeding challenges are associated with malnutrition
Source: Food Sci Nutr. 2023 Jun 12;11(9):5129–36. doi: 10.1002/fsn3.3472 (PMC10494642; doi:10.1002/fsn3.3472)
Supplement: Supplementary file 1 — Table S1 [file FSN3-11-5129-s001.docx]

**Table S1: Bivariate analysis of child feeding factors and nutrition status of children**

| Characteristics |  | Stunting |  |  | Wasting |  |  | Underweight |  |
| --- | --- | --- | --- | --- | --- | --- | --- | --- | --- |
|  | Normal  N (%) | Stunted  N (%) | Test Statistic | Normal  N (%) | Wasting  N (%) | Test Statistic | Normal  N (%) | Underweight  N (%) | Test Statistic |
| Age of children |  |  |  |  |  |  |  |  |  |
| 6-8 | 116 (87.2) | 17 (12.8) | **X2=24.553,**  **P=<0.001**** | 115 (86.5) | 18 (13.5) | **X2=5.447,**  **P=0.066**** | 114 (85.7) | 19 (14.3) | **X2=4.840,**  **P=0.089**** |
| 9-11 | 77 (90.6) | 8 (9.4) |  | 71 (83.5) | 14 (16.5) |  | 79 (92.9) | 6 (7.1) |  |
| 12-23 | 127 (68.6) | 58 (31.4) |  | 171 (92.4) | 14 (7.6) |  | 171 (92.4) | 14 (7.6) |  |
| Place of delivery |  |  |  |  |  |  |  |  |  |
| Home | 21(30.9) | 47 (69.1) | **X2=5.293,**  **P=0.031**** | 64 (94.1) | 4 (5.9) | X2=2.476,  P=0.116 | 60 (88.2) | 8 (11.8) | X2=0.408,  P=0.523 |
| Health facility | 273 (81.5) | 62 (18.5) |  | 293 (87.5) | 42 (12.5) |  | 304 (90.7) | 31 (9.3) |  |
| Early initiation of breastfeeding |  |  |  |  |  |  |  |  |  |
| Within 1 hour of delivery | 253 (92.0) | 71 (21.9) | **X2=1.756,**  **P=0.185**** | 298 (92.0) | 26 (8.0) | **X2=18.781,**  **P=<0.001**** | 296 (91.4) | 28 (8.6) | **X2=2.027**  **P=0.154**** |
| After 1 hour of delivery | 67 (84.8) | 12 (15.2) |  | 59 (74.7) | 20 (25.3) |  | 68 (86.1) | 11 (13.9) |  |
| What was done with Colostrum? |  |  |  |  |  |  |  |  |  |
| Gave it to baby | 318 (79.5) | 82 (20.5) | *P=0.500* | 354 (88.5) | 46 (11.5) | *P=1.00** | 361 (90.3) | 39 (9.7) | *P=1.00** |
| Discarded it | 2 (66.7) | 1 (33.3) |  | 3 (100) | 0 |  | 3 (100) | 0 |  |
|  |  |  |  |  |  |  |  |  |  |
| Continued breastfeeding at 1 year |  |  |  |  |  |  |  |  |  |
| Yes | 306 (81.2) | 71 (18.8) | **X=11.102,**  **P=0.001**** | 334 (88.6) | 43 (11.4) | X=0.000  P=0.984 | 341 (90.5) | 36 (9.5) | X=0.110  P=0.740 |
| No | 14 (53.8) | 12 (46.2) |  | 23 (88.5) | 3 (11.5) |  | 23 (88.5) | 3 (0) |  |
| Food introduced before breastmilk |  |  |  |  |  |  |  |  |  |
| Nothing | 314 (79.5) | 81 (20.5) | *P=0.671** | 353 (89.4) | 42 (10.6) | *P=0.007** | 357 (90.4) | 38 (9.6) | *P=0.560** |
| Infant formula and other foods | 6 (75.0) | 2 (25.0) |  | 4 (50.0) | 4 (50.0) |  | 7 (87.5) | 1 (12.5) |  |
| Introduction of solid and semi-solid food at 6 months |  |  |  |  |  |  |  |  |  |
| Yes | 250 (77.9) | 71 (22.1) | **X2=2.237,**  **P=0.135**** | 285 (88.8) | 36 (11.2) | X2=0.062,  P=0.803 | 296 (92.2) | 25 (7.8) | **X2=6.442**  **P=0.011**** |
| No | 70 (85.4) | 12 (14.6) |  | 72 (87.8) | 10 (12.2) |  | 68 (82.9) | 14 (17.1) |  |
| Experienced feeding challenges |  |  |  |  |  |  |  |  |  |
| Yes | 220 (81.8) | 4 9 (18.2) | **X2=2.802,**  **P=0.094**** | 238 (88.5) | 31 (11.5) | X2=0.010,  P=0.922 | 246 (91.4) | 23(8.6) | X2=1.176  P=0.278 |
| No | 100 (74.6) | 34 (25.4) |  | 119 (88.8) | 15 (11.2) |  | 118 (88.1) | 16 (11.9) |  |
| Bottle Feeding |  |  |  |  |  |  |  |  |  |
| Yes | 56(86.2) | 9 (13.8) | **X2=2.159**  **P=0.180**** | 50 (76.9) | 15(23.1) | **X2=10.425**  **P=0.001**** | 57 (88.7) | 8 (12.3) | X2=0.613  P=0.434 |
| No | 264 (78.1) | 74 (21.9) |  | 307 (90.8) | 31 (9.2) |  | 307 (90.8) | 31 (9.2) |  |
| Minimum dietary diversity of children |  |  |  |  |  |  |  |  |  |
| ≥ 4 Food groups | 136 (77.3) | 40 (22.7) | X2=0.863,  P=0.351 | 156 (88.6) | 2 0(11.4) | X2=0.001,  P=0.977 | 162 (92.0) | 14 (8.0) | X2=1.061  P=0.303 |
| < 4 Food groups | 184 (81.1) | 43 (18.9) |  | 201 (88.5) | 26 (11.5) |  | 202 (202) | 25 (11.0) |  |
| Minimum meal frequency of children |  |  |  |  |  |  |  |  |  |
| Yes | 117(79.0 0 | 47 (21.0) | X2=0.046,  P=0.830 | 199 (88.8) | 25(11.2) | X2=0.032,  P=0.858 | 207 (92.4) | 17 (7.6) | **X2=2.516**  **P=0.113**** |
| No | 143 (79.9) | 36 (20.1) |  | 158 (88.3) | 21 (11.7) |  | 157 (87.7) | 22 (12.3) |  |
| Minimum acceptable diet of children |  |  |  |  |  |  |  |  |  |
| Yes | 114 (78.6) | 31 (21.4) | X2=0.085,  P=0.771 | 129 (89) | 16 (11.0) | X2=0.032,  P=0.857 | 133 (91.7) | 12(8.3) | X2=0.509  P=0.476 |
| No | 206 (79.8) | 52 (20.2) |  | 228 (88.4) | 30 (11.6) |  | 231 (89.5) | 27 (10.5) |  |

^[[1]](#footnote-1)^ * are P-values from Fisher exact test and used for comparison purpose only

^[[2]](#footnote-2)^ ****** were considered for multivariable regression analysis

1. *Italicized cells* are P-values from Fisher exact test and used for comparison purpose only [↑](#footnote-ref-1)
2. **Bold cells** were considered for multivariable regression analysis [↑](#footnote-ref-2)
